# The Intersection of the Genetic Architectures of Orofacial Clefts and Normal Facial Variation

**DOI:** 10.3389/fgene.2021.626403

**Published:** 2021-02-22

**Authors:** Karlijne Indencleef, Hanne Hoskens, Myoung Keun Lee, Julie D. White, Chenxing Liu, Ryan J. Eller, Sahin Naqvi, George L. Wehby, Lina M. Moreno Uribe, Jacqueline T. Hecht, Ross E. Long, Kaare Christensen, Frederic W. Deleyiannis, Susan Walsh, Mark D. Shriver, Stephen Richmond, Joanna Wysocka, Hilde Peeters, John R. Shaffer, Mary L. Marazita, Greet Hens, Seth M. Weinberg, Peter Claes

**Affiliations:** ^1^Department of Electrical Engineering, ESAT/PSI, KU Leuven, Leuven, Belgium; ^2^Medical Imaging Research Center, UZ Leuven, Leuven, Belgium; ^3^Department of Human Genetics, KU Leuven, Leuven, Belgium; ^4^Department of Oral Biology, Center for Craniofacial and Dental Genetics, University of Pittsburgh, Pittsburgh, PA, United States; ^5^Department of Anthropology, Pennsylvania State University, State College, PA, United States; ^6^Department of Biology, Indiana University Purdue University Indianapolis, Indianapolis, IN, United States; ^7^Department of Chemical and Systems Biology, Stanford University School of Medicine, Stanford, CA, United States; ^8^Department of Genetics, Stanford University School of Medicine, Stanford, CA, United States; ^9^Department of Health Management and Policy, College of Public Health, University of Iowa, Iowa City, IA, United States; ^10^Department of Orthodontics & The Iowa Institute for Oral Health Research, College of Dentistry, University of Iowa, Iowa City, IA, United States; ^11^Department of Pediatrics, McGovern Medical School and School of Dentistry, UT Health at Houston, Houston, TX, United States; ^12^Lancaster Cleft Palate Clinic, Lancaster, PA, United States; ^13^Department of Epidemiology, Institute of Public Health, University of Southern Denmark, Odense, Denmark; ^14^UCHealth Medical Group, Colorado Springs, CO, United States; ^15^Applied Clinical Research and Public Health, School of Dentistry, Cardiff University, Cardiff, United Kingdom; ^16^Department of Developmental Biology, Stanford University School of Medicine, Stanford, CA, United States; ^17^Department of Human Genetics, University of Pittsburgh, Pittsburgh, PA, United States; ^18^Department of Otorhinolaryngology, KU Leuven, Leuven, Belgium; ^19^Department of Anthropology, University of Pittsburgh, Pittsburgh, PA, United States

**Keywords:** NSCL/P, endophenotype, genetics, facial morphology, ALSPAC

## Abstract

Unaffected relatives of individuals with non-syndromic cleft lip with or without cleft palate (NSCL/P) show distinctive facial features. The presence of this facial endophenotype is potentially an expression of underlying genetic susceptibility to NSCL/P in the larger unselected population. To explore this hypothesis, we first partitioned the face into 63 partially overlapping regions representing global-to-local facial morphology and then defined endophenotypic traits by contrasting the 3D facial images from 264 unaffected parents of individuals with NSCL/P versus 3,171 controls. We observed distinct facial features between parents and controls across 59 global-to-local facial segments at nominal significance (*p* ≤ 0.05) and 52 segments at Bonferroni corrected significance (*p* < 1.2 × 10^–3^), respectively. Next, we quantified these distinct facial features as univariate traits in another dataset of 8,246 unaffected European individuals and performed a genome-wide association study. We identified 29 independent genetic loci that were associated (*p* < 5 × 10^–8^) with at least one of the tested endophenotypic traits, and nine genetic loci also passed the study-wide threshold (*p* < 8.47 × 10^–10^). Of the 29 loci, 22 were in proximity of loci previously associated with normal facial variation, 18 were near genes that show strong evidence in orofacial clefting (OFC), and another 10 showed some evidence in OFC. Additionally, polygenic risk scores for NSCL/P showed associations with the endophenotypic traits. This study thus supports the hypothesis of a shared genetic architecture of normal facial development and OFC.

## Introduction

Orofacial clefting (OFC) is one of the most frequent congenital malformations with a substantial functional, financial, and mental health burden to the persons and families affected (Wehby and Cassell, 2010). Non-syndromic cleft lip with or without cleft palate (NSCL/P) has a multifactorial etiology, with both environmental and genetic factors playing roles (Mangold et al., 2011). To date, approximately 40 genetic risk loci have been identified for non-syndromic clefts through genome-wide association studies (GWAS; Thieme and Ludwig, 2017). Despite the substantial number of loci discovered, the sample sizes in these studies are relatively low when compared to GWA-studies of schizophrenia (Schizophrenia Working Group of the Psychiatric Genomics Consortium, 2014) or height (Wood et al., 2014), which include over 100K individuals. Therefore, it is likely that additional NSCL/P risk loci are yet to be discovered. Furthermore, a thorough understanding of the roles that these loci play in development, and thus the etiology of OFC, is still lacking.

A comprehensive approach to studying the etiology of NSCL/P requires investigations from different angles. One approach is to study subclinical phenotypes that exist within the range of normal development and are associated with OFC. Such traits are referred to as endophenotypes, which can be present in the biological relatives of individuals with NSCL/P and are potentially an incomplete expression of susceptibility loci for OFC (Gottesman and Gould, 2003; Weinberg et al., 2006). A number of endophenotypes have been described for NSCL/P, for example, a mild disruption of the upper lip musculature (Neiswanger et al., 2007), the appearance of “whorls” on the surface of the lower lip (Neiswanger et al., 2009), dental anomalies (Howe et al., 2015), and altered facial shape. In particular, unaffected relatives have been shown to display midfacial retrusion and a broadening of the upper face versus comparison control groups (Weinberg et al., 2009; Roosenboom et al., 2015).

Extensive characterization of the NSCL/P facial endophenotype allows us to investigate whether the presence of facial endophenotypes indicates heightened genetic susceptibility for NSCL/P. The possibility that NSCL/P risk variants influence normal-range facial variation raises interesting questions about the intersection of the genetic architecture between normal and dysmorphic facial features. Some NSCL/P candidate genes have already been shown to impact normal facial morphology. Boehringer et al. (2011) found associations between two genetic loci, rs1258763 and rs987525, involved in NSCL/P and normal craniofacial traits, namely nose width and bizygomatic distance, respectively. In previous work, we found that six known NSCL/P risk variants affected nose, chin, eyebrow ridge, and philtrum 3D shape in a large unselected population (Indencleef et al., 2018). Moreover, in a recent GWAS of normal-range facial morphology in Europeans, there was strong evidence of enrichment for genes implicated in OFC and palate development (Claes et al., 2018).

In this study, we investigate the intersection of the genetic architectures of normal facial variation and NSCL/P, starting from explicitly defined NSCL/P facial endophenotypes. First, we quantify the NSCL/P facial endophenotype at multiple levels of scale, modeling both global and local facial shape variations in a sample of unaffected parents of individuals with NSCL/P (Claes et al., 2018). Next, we identify genetic loci affecting these discovered facial endophenotypic traits in a GWAS of individuals from the general population. Finally, we examine the extent to which the genetic architecture of the facial endophenotypic traits reflects a heightened susceptibility for OFC.

## Materials and Methods

### Sample and Recruitment Details

This study involved two datasets, each consisting of two cohorts. The EURO dataset contained 3D images and genome-wide genotype data collected from the United States and United Kingdom. These are individuals of European ancestry recruited for studies of normal-range facial variation. The PARENT dataset contained 3D images of unaffected parents of individuals with NSCL/P and a group of unselected individuals of normal-range facial variation (unaffected but no further exclusion based on a family history in OFC). An overview of the sample sizes is given in Table 1.

**TABLE 1:** Overview of used datasets.

Dataset Name	Cohorts	Sample sizes	Data	Descent
PARENT	Unaffected NSCL/P parents	264	3D images	Self-reported European
	Unselected individuals	3,171		
EURO	United States	4,680	3D images and genotype data	European
	United Kingdom	3,566		

The United States samples in the EURO dataset originated from the 3D Facial Norms cohort (3DFN) and studies at the Pennsylvania State University (PSU) and Indiana University-Purdue University Indianapolis (IUPUI). The United Kingdom dataset included samples from the Avon Longitudinal Study of Parents and their Children (ALSPAC). Institutional review board (IRB) approval was obtained at each recruitment site, and all participants gave their written informed consent prior to participation. For children, written consent was obtained from a parent or legal guardian. The individuals of the EURO dataset have been tested for associations with normal-range facial morphology in previous work (Claes et al., 2018; White et al., 2020a). In all datasets, participants with missing information on sex, age, height, weight, or with insufficient image quality were removed.

For the 3DFN sample, 3D images and genotype data were obtained from the 3D Facial Norms repository (Weinberg et al., 2016). The repository includes 3D facial surface images and self-reported demographic descriptors as well as basic anthropometric measurements from individuals recruited at four United States sites: Pittsburgh, PA (PITT IRB PRO09060553 and RB0405013); Seattle, WA (Seattle Children’s IRB 12107); Houston, TX (UT Health Committee for the Protection of Human Subjects HSC-DB-09-0508); and Iowa City, IA (University of Iowa Human Subjects Office IRB 200912764 and 200710721). Recruitment was limited to individuals aged 3–40 years old and of self-reported European ancestry. Individuals were excluded if they reported a personal or family history of any birth defect or syndrome affecting the head or face, a personal history of any significant facial trauma or facial surgery, or any medical condition that might alter the structure of the face. The intersection of unrelated participants with quality-controlled images, covariates, and genotype data from individuals of European descent resulted in 1,906 individuals for analysis.

The PSU sample included 3D images and genotypes of participants recruited through several studies at the Pennsylvania State University and sampled at the following locations: Urbana-Champaign, IL (PSU IRB 13103); New York, NY (PSU IRB 45727); Cincinnati, OH (UC IRB 2015-3073); Twinsburg, OH (PSU IRB 2503); State College, PA (PSU IRB 44929 and 4320); Austin, TX (PSU IRB 44929); and San Antonio, TX (PSU IRB 1278). Participants self-reported information on age, ethnicity, ancestry, and body characteristics, and data were gathered on height and weight. Individuals were excluded from the analysis if they were below 18 years of age and if they reported a personal history of significant trauma or facial surgery, or any medical condition that might alter the structure of the face. No restriction on ancestry or ethnicity was imposed during recruitment, but only individuals of European descent were used in this study. The intersection of unrelated European participants with quality-controlled images, covariates, and genotype data resulted in 1,990 individuals for analysis.

The IUPUI sample includes 3D images and genotypic data from individuals recruited in Indianapolis, IN and Twinsburg, OH (IUPUI IRB 1409306349). Participants self-reported information on age, height, weight, and ancestry at the time of the collection. Individuals who were below 18 years of age were included if they had a parent or legal guardian’s signature. No restrictions were placed on the recruitment of participants, but only individuals of European descent and those meeting all quality control criteria were used in this study (*n* = 784).

The United Kingdom sample was derived from the ALSPAC dataset, a longitudinal birth cohort in which pregnant women residing in Avon with an expected delivery date between April 1, 1991 and December 31, 1992 were recruited (Boyd et al., 2013; Fraser et al., 2013). At the time, 14,541 pregnant women were recruited and DNA samples were collected for 11,343 children. Genome-wide data was available for 8,952 subjects of the B2261 study, titled “Exploring distinctive facial features and their association with known candidate variants.” In addition to this, 4,731 3D images were available along with information on sex, age, weight, height, ancestry, and other body characteristics. The ALSPAC study website contains details of all the data that is available through a fully searchable data dictionary and variable search tool (link available in the Web resources section). Image quality control analysis resulted in the removal of 14 images of poor quality, 199 participants were removed due to self-reported non-European ancestry, 168 participants were removed because of missing covariate data and 726 individuals were removed because of relatedness. The intersection of participants of European ancestry with quality-controlled images, covariates, and genotype data included 3,566 individuals. Ethical approval for the study was obtained from the ALSPAC Ethics and Law Committee and the Local Research Ethics Committees. Informed consent for the use of data collected via questionnaires and clinics was obtained from participants following the recommendations of the ALSPAC Ethics and Law Committee at the time. Consent for biological samples has been collected in accordance with the Human Tissue Act (2004).

Unaffected parents in the PARENT dataset were recruited as part of the Pittsburgh Orofacial Cleft (POFC) project, an international multi-center effort focused on understanding the etiology of non-syndromic OFC (Weinberg et al., 2006). The POFC project recruits individuals with clefts, their family members, and population-based controls for extensive phenotypic and genomic analyses. IRB approval was obtained at each recruitment site, and all participants gave their written informed consent prior to participation (PITT IRB approvals: STUDY19080127 and CR19030367-003). As part of the phenotyping protocol, 3D facial surface images were captured. Only unaffected (i.e., without OFC) parents of children with a non-syndromic form of cleft lip with or without cleft palate and of European descent were included in the current study. The unaffected parents were identified through index cases (affected probands) served by local Craniofacial Centers or from birth defects registries in the United States and Denmark. After image quality control and only selecting individuals with information on sex, age, weight, and height, 264 unaffected parents were included in the analysis.

The group of unselected individuals (to serve as controls) in the PARENT dataset was recruited either as part of normal-range facial variation projects (EURO US Cohort described above) or as controls for the POFC project. There was therefore significant overlap between our unselected sample and the EURO US cohort. In all cases, individuals in this group were excluded if they reported a personal or family history of any birth defect or syndrome affecting the head or face, a personal history of any significant facial trauma or facial surgery, or any medical condition that might alter the structure of the face. Starting from 4,762 individuals, we selected for 3D images acquired with the 3dMDface camera system to avoid camera bias (White et al., 2020b), and selected individuals older than 21, since the youngest parent in the dataset was 22 at the time of image acquisition. This selection resulted in 3,171 individuals.

### Genotyping and Imputation

Due to the several genotyping platforms used for the EURO US cohort (details in White et al., 2020a), we chose to impute the samples from each platform separately and then combine the imputed results (Verma et al., 2014). For each dataset, standard data cleaning and quality assurance practices were performed based on the GRCh37 genome assembly. Phasing was performed using SHAPEIT2 (v2.r900) (Delaneau et al., 2013) and imputation to the 1000G Phase 3 reference panel (The 1000 Genomes Project Consortium, 2015) performed using the positional Burrows-Wheeler Transform (Durbin, 2014) pipeline (v3.1) of the Sanger Imputation Server (v0.0.6) (McCarthy et al., 2016). After post-imputation quality control and intersection of imputed SNPs, a single merged dataset of all United States participants was created with 7,417,619 SNPs for analysis.

The raw genotype data from the EURO UK cohort (ALSPAC) was not available and restrictions are in place against merging the ALSPAC genotypes with any others. For this reason, ALSPAC genotypes, phased using SHAPEIT2 (Delaneau et al., 2013) and imputed to the 1000G Phase 1 reference panel (Version 3) (1000 Genomes Project Consortium et al., 2012) using IMPUTE2 (Howie et al., 2011), were obtained directly from the ALSPAC database and held separately during the analysis. After post-imputation quality control, the ALSPAC dataset contained 8,629,873 SNPs for analysis.

For both datasets, SNPs on the X chromosome were coded 0/2 for hemizygous males, to match with the 0/1/2 coding for females (Shaffer et al., 2016).

### Image Processing

The 3D facial images were obtained with digital facial stereophotogrammetry systems or laser scanning systems. For the 3DFN sample and the PARENT dataset, 3D images were acquired using the 3dMDface (3dMD, Atlanta, GA, United States) camera system. PSU sample images were obtained with either the 3dMDface or Vectra H1 (Canfield Scientific, Parsippany, NJ, United States) systems. The IUPUI sample was also imaged using the Vectra H1 system. The ALSPAC sample was imaged using a Konica Minolta Vivid 900 laser scanner (Konica Minolta Sensing Europe, Milton Keynes, United Kingdom). All participants were asked to have their mouths closed and to maintain a neutral facial expression during image capture.

3D surface images of both the EURO and PARENT dataset were imported into Matlab 2017b to perform the spatially dense registration process in the MeshMonk registration framework (White et al., 2019). Five positioning landmarks were roughly indicated to establish a crude alignment of the images. Then, each image was cleaned to remove hair, ears, and any dissociated polygons. MeshMonk was then used to map a symmetric (relative to the sagittal plane) anthropometric mask of 7,160 landmarks onto the images and their reflections, which were constructed by changing the sign of the *x* coordinate. This process resulted in homologous configurations of spatially dense quasi-landmarks for all images. Subsequently the original and reflected images were aligned using Generalized Procrustes Analysis (GPA) to eliminate differences in position, orientation and scale. Because we only wish to study variation in symmetric facial shape in this work, the original and reflected quasi-landmark configurations were averaged and the resulting symmetric configurations were used in further analyses.

After the registration, image quality control was performed to identify poorly remapped faces using two approaches. First, as described in Claes et al. (2018), outlier faces were identified by calculating Z-scores from the Mahalanobis distance between the cohort average and each individual face. Faces with Z-scores higher than two were manually checked. Second, a score was calculated that reflects the missing data present in the image due to holes, spikes, and other mesh artifacts that can be caused by facial hair or errors during the preprocessing steps. Images with scores indicating a high amount of missing data were also manually checked. During the manual check, the images were either classified as images of poor quality or were preprocessed and mapped again.

### Global-to-Local Facial Phenotyping

**FIGURE 1:**
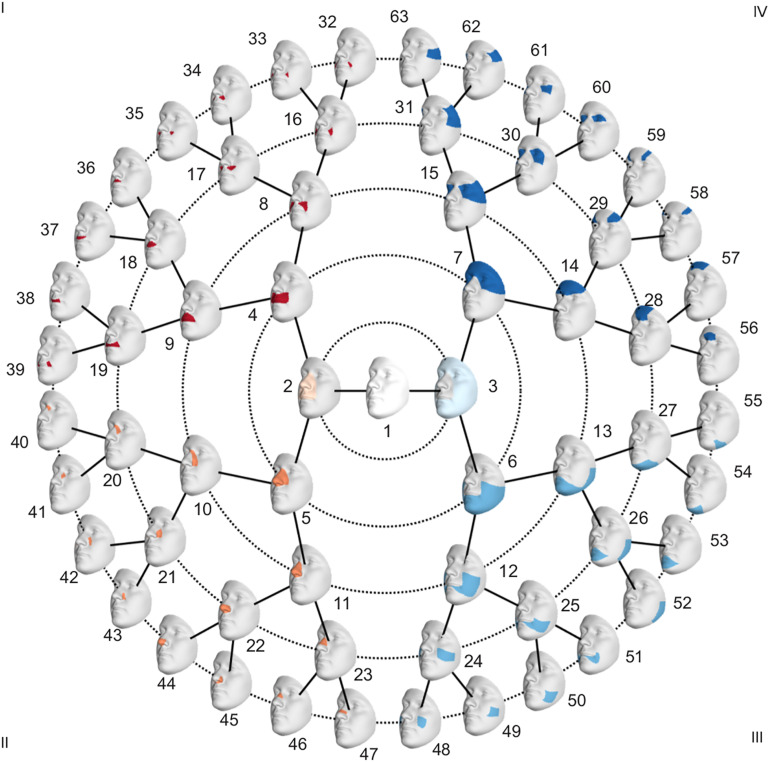
Global to local facial segmentation obtained using hierarchical spectral clustering.

To investigate facial shape on a global as well as a local scale, a data-driven facial segmentation was performed in the EURO dataset (Claes et al., 2018). Before segmentation, shape variables were adjusted for sex, age, age-squared, height, weight, facial size, the first four genomic ancestry axes, and the camera system by removing their effect using PLSR (function plsregress from Matlab 2017b) in the United States cohort and United Kingdom cohort separately. After adjustment, the two cohorts were combined, and the facial segments were defined by grouping vertices that are strongly correlated using hierarchical spectral clustering. The strength of covariation between quasi-landmarks was defined using Escoufier’s RV coefficient (Klingenberg, 2009). The RV coefficient was then used to build a structural similarity matrix that defined the hierarchical construction of 63 facial segments, broken into five levels, shown in Figure 1. The configurations of each segment were then independently subjected to a GPA to eliminate differences in rotation and location, after which a PCA was performed in combination with parallel analysis to capture the major variance in the facial segments with fewer variables (Hayton et al., 2004).

### Global-to-Local Regression-Based Endophenotypes

To adjust for confounding variables in the PARENT dataset, effects of sex, age, height, weight, and facial size were removed using PLSR. After the segmentation in the EURO dataset, the faces in the PARENT dataset were subjected to the same segmentation, and their shapes were projected in the PCA space of each segment. This was possible because all images were mapped with the same facial template. Subsequently, the facial phenotypes of the parents and the unselected individuals were contrasted in each of the 63 segments (Figure 1) by performing a shape regression. Using PLSR, statistical significance was tested following a permutation framework in which the effect size (*R*^2^) was used as a test-statistic using 10,000 permutations. In each segment, the PLSR returned one direction of effect or trait, which is a vector in multidimensional shape space. Traits defined in segments in which a nominally significant difference between parents and unselected individuals was observed were selected (*p* < 0.05) for further analysis and will be referred to as endophenotypic traits. These endophenotypic traits are not independent due to the overlapping construction of the facial segments and the dependency between neighboring quasi-landmarks of the facial template used in the registration. A Bonferroni significance threshold (*p* < 1.2 × 10^–3^) was calculated as 0.05/41, taking into account the multiple testing of the effective number of independent traits. The number of independent traits, 41, was calculated from the eigenvalues of a correlation matrix containing the pairwise multivariate correlations of all 63 traits (Li and Ji, 2005). Next, we quantified the presence of each of the identified endophenotypic traits in the EURO dataset. For each segment, the endophenotypic trait of that segment and the individual’s 3D shape in that segment are points or vectors in a multidimensional PCA space (Supplementary Figure 1). We measured the cosine distance that operates on the angle between two multi-dimensional vectors, one vector being the endophenotypic trait and the other an individual’s face. The lower the cosine distance, the more the endophenotypic trait is present in the individual face. Therefore, the cosine distance is a univariate score of the presence of the endophenotypic trait in facial segments of an individual.

### Genome Wide Association Analysis

A GWAS meta-analysis was performed on the endophenotypic scores in the United States and United Kingdom cohorts. For all analyses, the genotypes were coded additively based on the presence of the major allele. In total, 7,417,619 SNPs overlapped in both the United States and the United Kingdom cohort and were tested for association. In both cohorts, the endophenotypic scores were tested for genotype–phenotype associations in a standard linear regression with the SNP genotype as the independent variable and the univariate endophenotypic scores as the dependent variable (function regstats in Matlab 2017b). This function employs a *t*-statistic. Finally, the associations in the United States and the United Kingdom cohort were meta-analyzed using inversed variance weighting (Begum et al., 2012). Lead SNPs (i.e., the SNP with the smallest association *p*-value for each locus) were annotated when at least genome-wide significance (*p* < 5 × 10^–8^) was reached. Adjacent SNPs were clumped within a 1 Mb window (±500 kb) as the same signal of the lead SNP. Although annotations were performed for loci based on a genome-wide threshold, the study-wide Bonferroni threshold based on 59 tested traits was calculated as 5 × 10^–8^/59 (*p* = 8.47 × 10^–10^). A lead SNP was defined to show strong evidence for a role in OFC genetics if either a gene in the vicinity (±500 kb) showed functional evidence or if evidence from multiple research domains were found: genetic association studies, animal studies, and/or clinical studies. Weaker evidence was annotated to lead SNPs in the following cases: suggestive associations with OFC, a single clinical case study, a role in craniofacial development but not specifically in OFC or associations in the same chromosomal band but more than 500 kb removed from the lead SNP.

### Polygenic Risk Scores for NSCL/P

To investigate whether the endophenotypic scores capture a heightened genetic susceptibility for NSCL/P in an approach complementary to the GWAS, we calculated polygenic risk scores for NSCL/P in the EURO dataset. To this end, the PRSice software (Choi and O’Reilly, 2019) was used to create a model from the summary statistics of a previously published NSCL/P meta-analysis (Leslie et al., 2017) based on individuals of European descent only (*N* = 1,411). Mismatched SNPs were removed by PRSice, and SNPs were clumped using an LD *r*^2^ threshold of 0.1 in a window of ±250 kb. Polygenic risk scores were calculated with inclusion *p*-value thresholds ranging from 5 × 10^–8^ to 1 with a step size of 5 × 10^–5^, resulting in 10^4^ sets of polygenic risk scores. Finally, for each inclusion threshold, an association between the endophenotypic scores (independent variable) and the polygenic risk scores (dependent variable) was tested using linear regression (function regstats in Matlab2019b). This was done for the United States and United Kingdom cohort separately.

## Results

**FIGURE 2:**
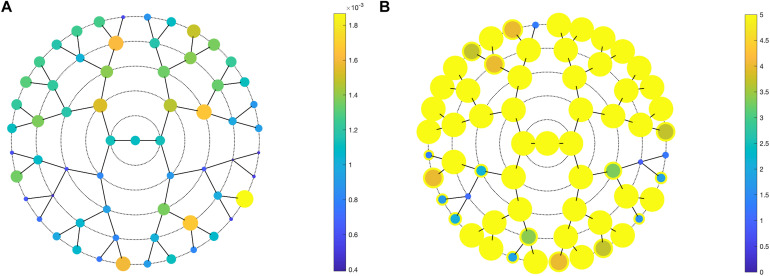
PLSR results. PLSR statistics of unaffected parents versus unselected individuals, showing **(A)** the effect size (*R*^2^) and **(B)** the -log10(*p*-values) in all 63 segments. **(A)** Larger circle size indicates a larger effect size or a lower *p*-value **(B)**. In panel **(B)**, a yellow outer circle indicates nominal significance in that segment (*p* < 0.05).

**FIGURE 3:**
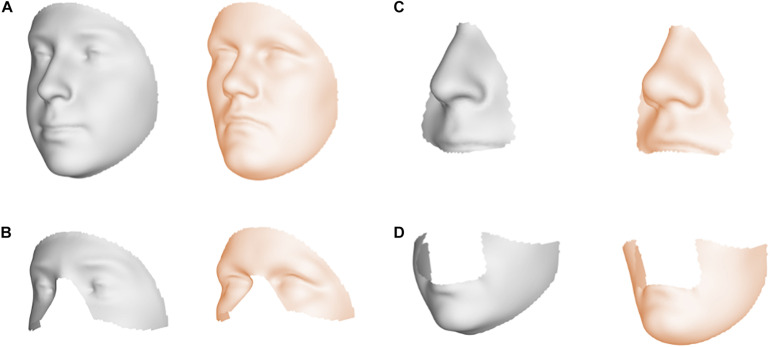
Endophenotypic traits. Visualization of the endophenotypic traits by exaggerating the traits in the direction of the control group (gray) and the NSCL/P relative group (orange). **(A)** Segment 1, **(B)** Segment 2, **(C)** Segment 6, and **(D)** Segment 7.

Facial shape in 59 out of 63 segments was found to be different between unaffected parents of individuals with NSCL/P and the unselected individuals at nominal significance (*p* < 0.05), as shown in Figure 2. In 52 segments, a significant difference was found below the Bonferroni-corrected threshold (*p* < 1.3 × 10^–3^). Each shape regression returned a direction of effect, represented by a vector in multidimensional shape space, which we refer to as the endophenotypic trait. Endophenotypic traits that were identified in segments 1, 2, 6, and 7 are shown in Figure 3, respectively, covering the full face, the midface, the mandibular region, and the upper facial region. These endophenotypic traits illustrate a midfacial retrusion and a broadening of the upper face, a more protruded chin, and an upturned nose. All 59 global-to-local traits are visualized in Supplementary Figure 2.

**FIGURE 4:**
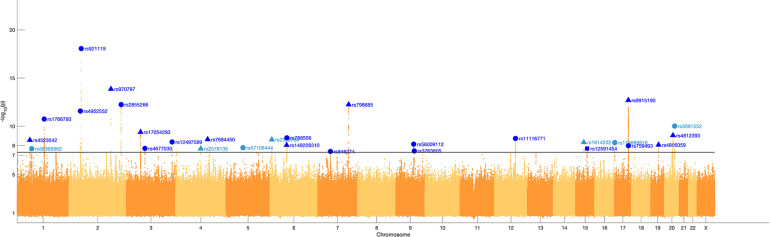
Manhattan plot. A Manhattan plot of the meta-analysis *p*-values of the endophenotypic traits GWAS. The horizontal line represents the genome-wide significance threshold (*p* < 5 × 10^–8^). The 29 lead SNPs are labeled with RS-numbers in dark blue if there is overlap with a GWAS on normal facial variation (White et al., 2020a), and light blue if there is no overlap. The triangle, circle, and square symbols mark strong, weak, and no evidence for a role of the lead SNP in orofacial clefting, respectively.

**FIGURE 5:**
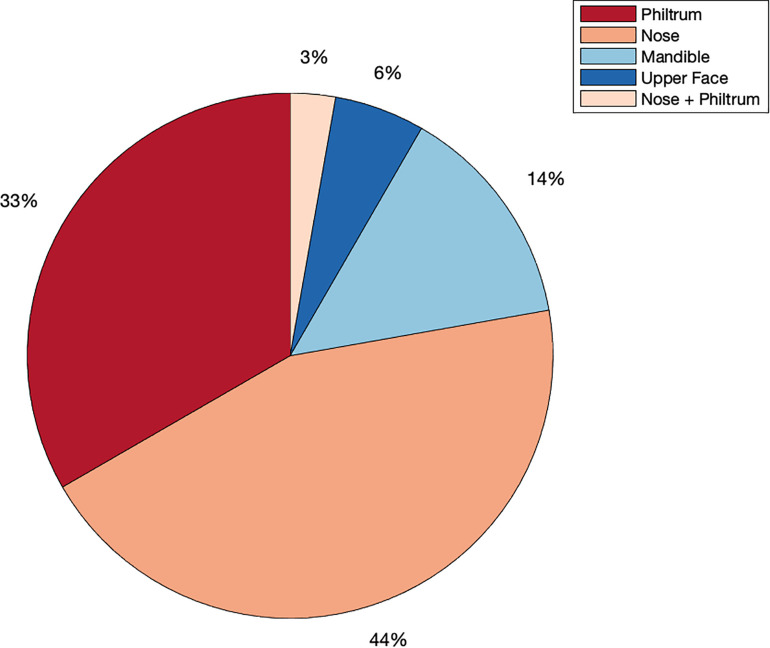
Meta-analysis phenotype hits. Percentage of all genome-wide associations (*p* < 5 × 10^–8^) that occurred in quadrant I or the philtrum quadrant (red), quadrant II or the nasal quadrant (orange), quadrant III or the mandibular quadrant (light blue), quadrant IV or the upper facial quadrant (dark blue) or in segment 2, which covers the nose and philtrum area (light orange).

We identified 29 independent genetic loci with a lead SNP associated with at least one of the 59 endophenotypic traits below a genome-wide significance threshold (*p* < 5 × 10^–8^) (Figure 4). In total, 36 associations were significant at a genome-wide level as some of the 29 lead SNPs were associated with more than one trait. Of these 36 associations, 16 included traits in quadrant II (nose, Figure 1), 12 in quadrant I (philtrum), 5 in quadrant III (mandible), 2 in quadrant IV (upper face), and lastly 1 association in segment 2 (midface, Figure 5). Nine out of 29 lead SNPs also passed the study-wide threshold (*p* < 8.47 × 10^–10^). SNP information and meta-analysis statistics of the 29 best associations (lowest *p*-value of all associated traits) are given in Table 2. Supplementary Table 1 contains additional information on the regression statistics and information for all 36 significant associations. Twenty-two of the lead SNPs lie in proximity (±500 kb) to lead SNPs identified in a prior normal facial variation GWAS (White et al., 2020a). Sixteen lead SNPs showed strong evidence for a role in risk for OFC, another 12 showed weaker evidence (Supplementary Table 2).

**TABLE 2:** Meta-analysis results.

RS	Chr	Pos	Locus	A1	A2	MAF US	MAF UK	Beta	SE	*p*-value	Segment
rs4523542	1	60996971	1p31.3	T	C	0.49	0.50	–0.0293	0.0052	1.53E-08	47
rs56399962	1	68159266	1p31.3	T	A	0.23	0.22	–0.0203	0.0036	2.09E-08	4
rs1766793	1	119466374	1p12	G	T	0.29	0.29	–0.0427	0.0063	1.76E-11	41
rs4952552	2	42198217	2p21	A	G	0.45	0.44	–0.0401	0.0057	2.65E-12	43
rs921119	2	45272812	2p21	C	T	0.29	0.30	0.0446	0.005	8.71E-19	34
rs970797	2	177111819	2q31.1	T	G	0.43	0.43	0.0467	0.0061	1.38E-14	33
rs2855266	2	223072019	2q36.1	A	T	0.14	0.15	0.0597	0.0083	5.59E-13	46
rs17054293	3	54724560	3p14.3	A	T	0.27	0.27	–0.0424	0.0068	4.02E-10	33
rs4677030	3	71230514	3p14.1	A	G	0.42	0.41	–0.0246	0.0044	2.00E-08	23
rs12497589	3	184464709	3q27.1	A	G	0.34	0.34	0.0193	0.0035	2.40E-08	24
rs2578135	4	94864577	4q22.2	T	C	0.48	0.47	0.0212	0.0038	2.12E-08	53
rs7694450	4	121998104	4q27	A	G	0.45	0.43	0.0298	0.005	2.32E-09	39
rs57106444	5	67765894	5q13.1	G	GTT	0.15	0.14	–0.0258	0.0046	1.67E-08	60
rs2326766	6	6393064	6p25.1	T	C	0.42	0.43	0.0272	0.0046	2.45E-09	37
rs149228310	6	51091163	6p12.3	G	A	0.39	0.38	–0.0251	0.0044	9.08E-09	45
rs788506	6	51703012	6p12.2	T	A	0.38	0.36	–0.023	0.004	8.80E-09	44
rs846274	7	42061986	7p13	A	G	0.43	0.44	–0.0232	0.0042	4.11E-08	45
rs798685	7	121985541	7q31.32	T	C	0.26	0.29	0.0341	0.0047	5.74E-13	45
rs56009112	9	94480903	9q22.31	A	G	0.31	0.32	–0.0148	0.0026	7.24E-09	2
rs3763605	9	96420390	9q22.32	A	G	0.34	0.34	0.032	0.0058	3.60E-08	16
rs11116771	12	85686488	12q21.32	T	C	0.35	0.35	0.0289	0.0048	1.81E-09	37
rs1614202	15	57716048	15q22.2	G	A	0.41	0.39	0.0247	0.0044	2.59E-08	59
rs12591454	15	71876010	15q24.1	A	G	0.33	0.33	–0.0359	0.0064	2.06E-08	33
rs113689916	17	1196526	17p13.3	T	C	0.02	0.01	–0.1408	0.0241	4.97E-09	35
rs9915190	17	69142628	17q25.1	A	C	0.37	0.41	–0.0434	0.0061	1.14E-12	40
rs759493	17	70036558	17q25.1	C	T	0.42	0.41	0.0308	0.0054	1.05E-08	47
rs4805059	19	34294901	19q12	A	G	0.48	0.47	–0.0307	0.0053	8.51E-09	47
rs4812393	20	38073575	20q12	T	C	0.46	0.48	0.0202	0.0033	8.96E-10	26
rs3091552	20	45440006	20q13.12	C	G	0.24	0.25	0.0351	0.0054	9.63E-11	45

*Lead SNPs reaching at least genome-wide significance (*p* < 5 × 10^–8^). For each SNP, this table contains the RS-number (RS), chromosome (Chr), position (Pos, GRCh37/hg19), cytogenetic band (Locus), minor allele (A1), major allele (A2), minor allele frequency in the United States cohort (MAF US), minor allele frequency in the United Kingdom cohort (MAF UK), meta-analysis regression coefficient (Beta), meta-analysis standard error (SE), meta-analysis *p*-value (*p*-value), and the segment in which the endophenotypic trait showing the highest association with the lead SNP was identified in (Segment).*

**FIGURE 6:**
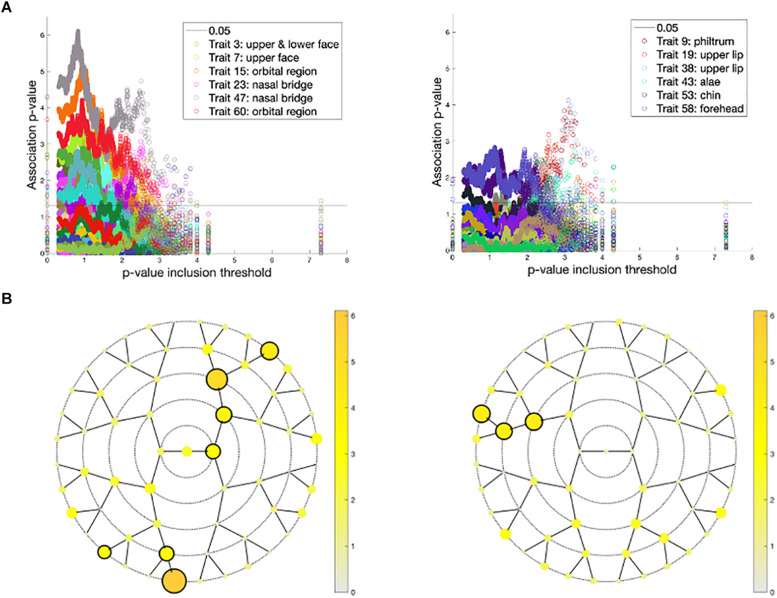
Associations between polygenic risk scores and endophenotypic scores. **(A)** The -log10 *p*-values of the association (*y*-axis) plotted against −log10(*p*-value inclusion thresholds) (*x*-axis) in the United States cohort **(left)** and the United Kingdom cohort **(right)**. For both cohorts, only the six strongest associated endophenotypic traits are depicted following the inset legends. The horizontal line represents a *p*-value of 0.05. **(B)** The minimal association −log10(*p*-value) across all *p*-value inclusion thresholds was plotted for each segment in the United States cohort **(left)** and the United Kingdom cohort **(right)**. Encircled segments show associations of *p*-value < 0.0013 (0.05/38 independent traits, calculated from the 59 traits tested).

Associations of the 59 endophenotypic traits with polygenic risk scores calculated across different *p*-value inclusion thresholds are visualized in Figure 6. In the United States cohort, the strongest associations occurred in polygenic risk scores calculated with inclusion thresholds around 0.1. The endophenotypic traits showing the strongest associations are present in the forehead, eye, and nose tip regions (Figure 6). The strongest associations in the United Kingdom cohort were found between the endophenotypic traits and polygenic risk scores calculated using a *p*-value inclusion threshold around 0.001. The traits responsible for these associations are localized in the philtrum area (Figure 6).

## Discussion

Orofacial clefting has a wide range of expression and severity (Marazita, 2012). CL/P can occur bilaterally or unilaterally and is generally considered distinct from cleft palate only (CPO), based on embryological and epidemiological considerations (Roosenboom et al., 2017). The range of severity also includes minor defects and subclinical phenotypes, which are on the mild side of the severity spectrum. One of those subclinical phenotypes includes subtle alterations in facial shape—a facial endophenotype indicative of NSCL/P risk. Unaffected members of families with a history of OFC have been found to show a broadening of the upper face and midfacial retrusion in comparison to control groups with no family history of clefting (Weinberg et al., 2009; Roosenboom et al., 2015, 2017). These endophenotypic features were confirmed in this work, where unaffected parents also showed an upturned nose and a protrusion of the chin.

Although several studies have confirmed the presence of a facial endophenotype for NSCL/P in unaffected relatives, a biological cause during embryonic development remains speculative. Lip formation happens around week 6 of gestation, and CL/P occurs when the nasal and maxillary prominences fail to fuse (Marazita, 2012). This could be the result of a failure of prominence growth and/or alignment due to the shape of the face and the direction of growth of the prominences. Mouse strains at higher risk for spontaneous clefting have been shown to exhibit unique patterns in the orientation of the facial prominences forming the lip and palate (Trasler, 1968; Juriloff and Trasler, 1976; Millicovsky et al., 1982; Young et al., 2007; Parsons et al., 2008). Mouse studies including mutants in *TFAP2A* [MIM: 107580], a gene implicated in CL/P, have suggested small gene expression changes altering the growth and shape of the facial prominences as a possible cause for the development of a cleft (Green et al., 2015). Accordingly, cleft risk variants might influence the shape of embryological structures in a way that complicates proper alignment for fusion. If fusion succeeds however, the altered shape could result in the facial endophenotype. This idea is supported by our endophenotype GWAS, in which the lead SNPs were predominantly associated with traits in the nasal and philtrum area (80% of the genome-wide associations were with traits in this area), the regions developed from the nasal and maxillary prominences. Thus, there is a clear intersection between normal facial development and shape alterations potentially leading to malformations such as CL/P.

In our GWAS of endophenotypes, 22 of the 29 lead SNPs among associated loci were found in vicinity (±500 kb) of the lead SNPs from a previous GWAS of normal facial variation (White et al., 2020a). The overlap of the endophenotypes with normal facial variation is substantial and expected. The endophenotypic traits were established by analyzing unaffected parents’ facial shape, yet tested in a sample of normal facial variation where a family history of cleft was absent or unknown. An intersection between the genetic pathways of NSCL/P and normal facial variation was already implicated by the influence of NSCL/P risk variants on normal facial variation. Boehringer et al. found associations between two NSCL/P loci and bizygomatic distance and nose width (Boehringer et al., 2011). In previous work, we found six NSCL/P risk variants affecting nose, chin, eyebrow ridge, and philtrum 3D shape in the larger unselected population (Indencleef et al., 2018). One of those six variants, rs6740960, has been shown to play a role in both NSCL/P and normal facial variation in multiple studies. In a normal facial variation GWAS conducted by our group, rs6740960 was associated with shape in the mandibular area (Claes et al., 2018). The same variant was associated with NSCL/P in a multi-ethnic meta-analysis (Ludwig et al., 2017). In this work, rs4952552 (17 kb from rs6740960) was associated with segment 43, which covers small regions on the outer sides of the nostrils. Our results indicate a strong overlap in the underlying genetics of the NSCL/P endophenotype and normal facial variation, with rs6740960 as an example of this intersection.

The facial endophenotype is hypothesized to be an expression of NSCL/P susceptibility genes. In this work, 16 of the 29 lead SNPs also have substantial evidence for a role in OFC and 12 other lead SNPs show weaker evidence (Supplementary Table 2). Of special interest are the seven lead SNPs implicated with OFC that did not show an overlap with normal facial variation, which will be discussed here. One of these variants is rs56399962, an SNP located in 1p31.3. A clinical report covering a patient with a deletion in 1p31 described a unique form of OFC manifesting as a facial midline defect in both the lower and upper lip (Y1ld1r1m et al., 2014). In a family based study, rs787499, an SNP 87 kb from rs56399962, was associated with NSCL/P (Camargo et al., 2012). This variant is also 404 kb from *WLS* [MIM: 611514], of which expressed mRNA and protein have been found in the embryonic development of the lip and palate in mice (Yu et al., 2010). *WLS* deletion in mice has resulted in abnormal palate development (Fu et al., 2011). In zebrafish, this gene was shown to be involved in palate morphogenesis (Rochard et al., 2016). These findings show that rs56399962 is located in a possible risk locus for NSCL/P, with *WLS* as a candidate gene. Another SNP associated with NSCL/P endophenotypes in our study, rs2578135, is 169 kb from a transcript variant of *GRID2* [MIM: 602368], a gene that showed suggestive evidence (*p*-value = 7.9 × 10^–7^) with NSCL/P in a GWAS with an Asian cohort (Beaty et al., 2010). *GRID2* has also shown evidence for a gene-smoking interaction effect for CL/P (Beaty et al., 2013). Another SNP, rs57106444, which is 250 kb from rs831227, showed a suggestive association (*p*-value = 5.34 × 10^–7^) in a POFC multi-ethnic GWAS for NSCL/P (Leslie et al., 2016). *PIK3R1* [MIM: 171833], a gene 168 kb from rs57106444, was differentially expressed in association with NSCL/P (Wang et al., 2016). *F13A1* [MIM: 134570] is a gene located 72 kb from rs2326766 in 6p25.1, which was associated with an endophenotypic trait in the philtrum. This gene was suggested as a candidate gene in a NSCL/P linkage study, but this result has not been replicated (Eiberg et al., 1987). Although 6p23 has been a candidate locus for NSCL/P in various studies, five individuals with isolated 6p25 deletions have been reported with a high or cleft palate (Carinci et al., n.d.; Scapoli et al., 1997; Linhares et al., 2015). The variant identified in 15q22.2 (rs2326766) lies in the same chromosomal band but still 5MB from a SNP that was suggestively associated with NSCL/P (de Aquino et al., 2014). Another variant, rs113689916, located in 17p13.3 and associated with an endophenotypic trait in the philtrum, lies within a microduplication site for which the syndromic phenotype shows CL/P (Curry et al., 2013; Tucker and Escobar, 2014; Ibitoye et al., 2015). Lastly, rs3091552 is associated with an endophenotypic trait in the nose and showed a suggestive association (*p* = 2.5 × 10^–5^) in a previous NSCL/P GWAS (Leslie et al., 2017). The SNP is located in 20q13.12, the duplication of which has caused cleft lip/palate in two clinical cases (Blanc et al., 2008). One familial case of Galloway-Mowat syndrome [MIM: 617730] due to a novel *TP53RK* [MIM: 608679] mutation, a gene within 500 kb of rs3091552, also showed a cleft palate (Hyun et al., 2018). Although these seven variants have not yet been significantly associated with either normal facial variation or NSCL/P in a GWAS, these results imply that they are new candidate risk variants for NSCL/P in a European population, with a potential additional role in normal facial variation.

The strong overlap of the endophenotypic lead SNPs and genetics of OFC indicates that the endophenotype is an expression of NSCL/P susceptibility genes and that our quantification method has captured this heightened susceptibility successfully. In further support of this observation, we have provided additional evidence by calculating polygenic risk scores for NSCL/P and subjecting them to an association with the endophenotypic traits. In both the United States and United Kingdom cohorts, endophenotypic traits showed associations with the polygenic risk scores, indicating an overall heightened susceptibility for NSCL/P. Although associations were found in both United States and United Kingdom cohorts, the specific traits associated were different. The United Kingdom cohort showed associations in the philtrum, an important area in lip formation during development. In the same cohort, Howe et al. (2018) found an association between philtrum width and an increased PRS for NSCL/P. These results indicate that variants affecting the event of lip fusion can also influence philtrum shape. The United States cohort showed associations in the orbital region and in the nasal tip. During embryological development, the nasal prominence is involved in lip formation. Even though the forehead region does not seem to be directly involved in lip formation, it has previously been identified as a region involved in the facial endophenotype (Weinberg et al., 2009; Roosenboom et al., 2015). The discordances of traits associated with the PRS scores between the United States and United Kingdom cohorts could be present due to a couple of reasons. First, in contrast to the United States, the United Kingdom cohort only includes individuals of 15 years old, which do not have fully developed facial features. Second, the summary statistics used to calculate the PRS are based on United States individuals. Although all are of European ancestry, genetic differences between populations of United States and United Kingdom are likely to exist and influence the polygenic risk scores predicted. Lastly, the sample sizes for calculating PRS are relatively small, and the summary statistics were based on a GWAS of *N* = 1,411 individuals.

In this work, we tested a range of inclusion thresholds for the polygenic risk scores. However, the common practice is to determine an optimal inclusion threshold in another independent dataset (Dudbridge, 2013), which was not available in this work. For most polygenic traits, the more the SNPs included in the polygenic risk model, the higher the predictive power (Dudbridge, 2013). This often leads to relatively high optimal inclusion thresholds such as *p* < 0.5. In a study by Ludwig et al. (2017), the optimum inclusion threshold for NSCL/P polygenic risk scores was *p* < 0.2. In contrast, Howe et al. (2018) found *P* < 10^–5^ to be the optimum inclusion *p*-value and thus included a lower number of SNPs. Consequently, the optimum *p*-value inclusion threshold seems to be strongly dependent on the trait as well as the datasets used. Linkage disequilibrium score regression is a method in which genetic correlations between different traits are estimated, yet unfortunately our sample sizes did not meet the criteria for this analysis.

In this study, we focused our analyses on individuals of European ancestry. To analyze possible population similarities and differences in both OFC genetics as well as normal facial genetics, future analyses should focus on individuals of other ancestries. For example in the context of NSCL/P GWA-studies, evidence for the gene desert 8q24 is consistently strong in European ancestry groups, but less so in Asian ancestry groups (Beaty et al., 2016). Inclusion of more diverse populations in human genetics studies is of great importance, because the current bias for European ancestry can translate into a poor disease prediction and treatment for individuals of under-represented ancestries (Sirugo et al., 2019). Another limitation of this study is the lack of additional datasets to replicate the genetic associations found, as well as the polygenic risk scores. More specifically in the PRS analysis, additional datasets would be most beneficial to identify the optimum *p*-value inclusion threshold. A third limitation is that the unselected individuals are extracted from different databases in which extensive filtering was necessary. Additionally, only the unselected individuals included from our collaboration with Pittsburgh were checked for a family history of OFC, and excluded if it was indeed so. Lastly, no functional analyses were performed to further investigate the role of the loci discovered in OFC. This would be of great interest for future analyses.

This study is the first to identify facial endophenotypic traits for NSCL/P based on a comprehensive global-to-local facial phenotyping approach. We have used these traits to examine their underlying genetic architecture and observed a substantial link between the endophenotypic traits and NSCL/P genetics, with 16 of our lead SNPs possessing strong literature evidence for an association with NSCL/P and an additional 12 SNPs having weaker literature evidence. We identified seven variants as possible new candidate risk loci for NSCL/P. Furthermore, endophenotypic traits also indicated an overall heightened NSCL/P susceptibility based on polygenic risk scores. This study delivers additional evidence for the existence of the facial endophenotype as an expression of NSCL/P susceptibility genes. Our study shows that 76% of our lead SNPs are also associated with normal facial variation. Therefore, and in conclusion, this study contributes to the understanding of the intersection of NSCL/P genetics and the genetic architecture of normal facial variation.

## Data Availability Statement

Publicly available datasets were analyzed in this study. The data can be found here: All of the genotypic markers for the 3DFN dataset are available to the research community through the dbGaP controlled-access repository (http://www.ncbi.nlm.nih.gov/gap) at accession #phs000929.v1.p1. The raw source data for the phenotypes—the 3D facial surface models in.obj format—are available through the FaceBase Consortium (https://www.facebase.org) at accession #FB00000491.01. Access to these 3D facial surface models requires proper institutional ethics approval and approval from the FaceBase data access committee. Additional details can be requested from SMW. The participants making up the PSU and IUPUI cohorts were not collected with broad data sharing consent. Given the highly identifiable nature of both facial and genomic information and unresolved issues regarding risk to participants, we opted for a more conservative approach to participant recruitment. Broad data sharing of the raw data from these collections would thus be in legal and ethical violation of the informed consent obtained from the participants. This restriction is not because of any personal or commercial interests. Additional details can be requested from MS and SW for the PSU and IUPUI datasets, respectively. The ALSPAC (United Kingdom) data will be made available to bona fide researchers on application to the ALSPAC Executive Committee (http://www.bris.ac.uk/alspac/researchers/data-access). Ethical approval for the study was obtained from the ALSPAC Ethics and Law Committee and the Local Research Ethics Committees. The ALSPAC study website contains details of all the data that are available through a fully searchable data dictionary and variable search tool: http://www.bris.ac.uk/alspac/researchers/our-data/.

## Ethics Statement

The studies involving human participants were reviewed and approved by ALSPAC Ethics and Law Committee. Written informed consent to participate in this study was provided by the participants’ legal guardian/next of kin. Written informed consent was obtained from the individual(s), and minor(s)’ legal guardian/next of kin, for the publication of any potentially identifiable images or data included in this article.

## Author Contributions

KI performed all analyses and wrote the first draft of the manuscript under the supervision of PC. PC, JS, SMW, and MM conceptualized the design of the study. JS, SMW, MM, RE, SW, JW, and MS organized and imputed the EURO data. ML, GW, LM, JH, RL, KC, FD, JS, MM, and SMW collected and curated the PARENT data. SR coordinated the collection of the ALSPAC images. SN, CL, ML, HH, and JW provided input throughout the analyses and the writing process. All authors contributed to manuscript revision, and read and approved the submitted version.

## Conflict of Interest

The authors declare that the research was conducted in the absence of any commercial or financial relationships that could be construed as a potential conflict of interest. The reviewer AR-M declared a past co-authorship with one of the authors, GH, to the handling editor
