# Supplementary material for: The Intersection of the Genetic Architectures of Orofacial Clefts and Normal Facial Variation
Source: Front Genet. 2021 Feb 22;12:626403. doi: 10.3389/fgene.2021.626403 (PMC7937973; doi:10.3389/fgene.2021.626403)
Supplement: Supplementary file 1 [file Data_Sheet_1.zip › Figure S1 & Supplementary Tables Captions.DOCX]

Supplementary Material

# Supplementary Figures and Tables

## Supplementary Figures


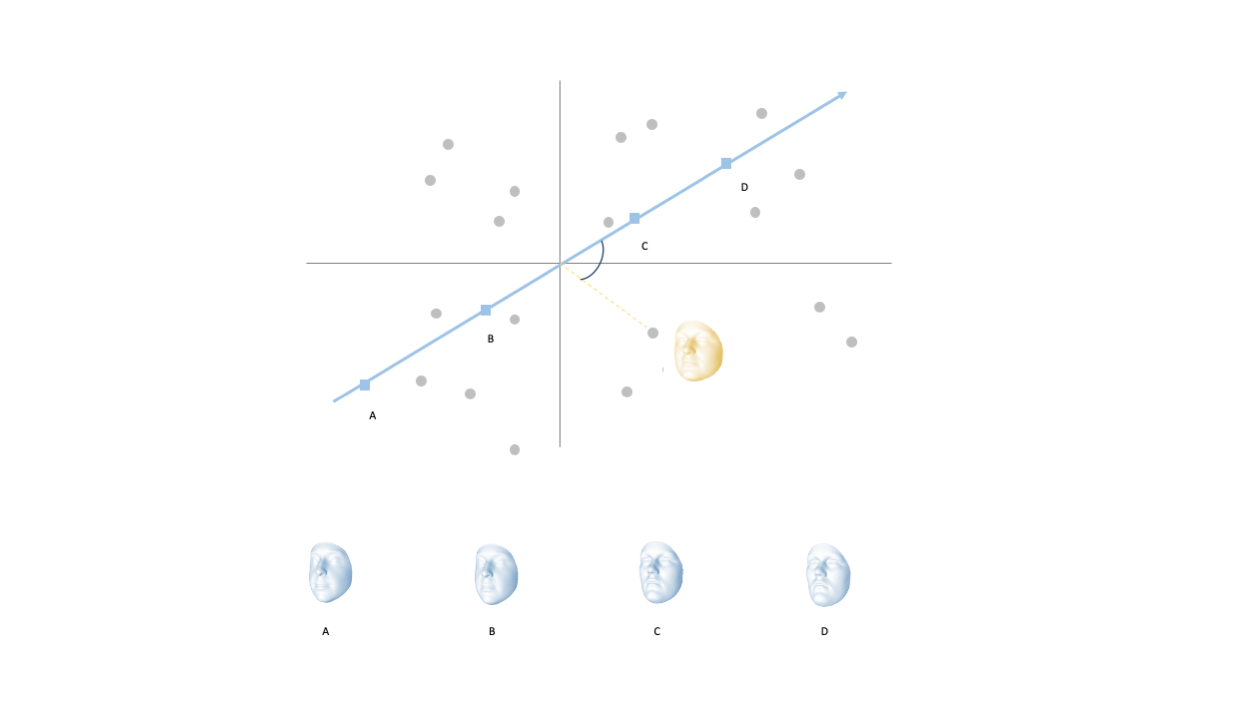


**Figure S1: Illustration of the quantification of the endophenotypic trait**. A 2D representation of a multi-dimensional shape PCA space, in which individual faces are represented as points in PCA space in grey. The solid blue arrow represents the endophenotypic trait identified in the partial least squares regression. The faces in the bottom (A-D) are visualizations of different points along the endophenotypic trait with D resembling the endophenotypic trait the most. An example of an individual face is displayed in yellow; the yellow dashed line represents its facial vector. The quantification of the endophenotypic trait for this face is performed by measuring the angle between the endophenotypic trait and the individual facial vector. This process is done for all individuals in the EURO dataset in each of the facial segments in Figure 2, for which nominal significance of the endophenotypic trait was obtained.

## Supplementary Tables

**Table S1 (*TableS1.xlsx*): GWAS and meta-analysis results for all associations.** Lead SNPs reaching at least genome-wide significance (p<5x10-8). For each association, this table contains the RS-number (RS), chromosome (Chr), position (Pos, GRCh37/hg19), minor allele (A1), major allele (A2), the segment in which the endophenotypic trait showing the highest association with the lead SNP was identified in (Segment), the quadrant the segment belongs to (Quadrant), minor allele frequency in the US cohort (MAF US), regression coefficient in the US cohort (Beta US), standard error in the US cohort (SE US), p-value in the US cohort (P US), minor allele frequency in the UK cohort (MAF UK), regression coefficient in the UK cohort (Beta UK), standard error in the UK cohort (SE UK), p-value in the UK cohort (P UK), meta-analysis regression coefficient (Beta Meta), meta-analysis standard error (SE Meta), meta-analysis p-value (P Meta).

**Table S2 (*TableS2.xlsx*): Literature evidence for a role in OFC for the lead SNPs.** For each lead SNP, this table contains the RS-number (RS), chromosome (Chr), position (Pos, GRCh37/hg19), cytogenetic band (Locus), a short description of the evidence for a role in OFC (Evidence), author of publication (Author), reference link (Reference), the last three columns indicate whether the lead SNP was considered to show strong or weak evidence for a role in clefting (the criteria for this annotation are discussed in section 4.2 - Genome wide association analysis), respectively, and lastly if the SNP is in proximity (± 500 kb) of a lead SNP of the normal facial variation GWAS.
