# Supplementary material for: The Intersection of the Genetic Architectures of Orofacial Clefts and Normal Facial Variation
Source: Front Genet. 2021 Feb 22;12:626403. doi: 10.3389/fgene.2021.626403 (PMC7937973; doi:10.3389/fgene.2021.626403)
Supplement: Supplementary file 1 [file Data_Sheet_1.zip › Figure S2.PDF]

Figure S2: Overview of the NSCL/P endophenotypic traits

K. Indencleef et al.

Contents

Segment 1 2
Segment 2 2
Segment 3 2
Segment 4 2
Segment 5 2
Segment 6 2
Segment 7 3
Segment 8 3
Segment 9 3
Segment 10 3
Segment 11 3
Segment 12 3
Segment 13 4
Segment 14 4
Segment 15 4
Segment 16 4
Segment 17 4
Segment 18 4
Segment 19 5
Segment 20 5
Segment 22 5
Segment 23 5
Segment 24 5
Segment 25 5
Segment 26 6
Segment 28 6
Segment 29 6
Segment 30 6
Segment 31 6
Segment 33 6
Segment 34 7
Segment 35 7
Segment 36 7
Segment 37 7
Segment 38 7
Segment 39 7
Segment 40 8
Segment 41 8
Segment 42 8
Segment 43 8
Segment 44 8
Segment 45 8
Segment 46 9
Segment 47 9
Segment 48 9
Segment 49 9
Segment 50 9
Segment 51 9
Segment 52 10
Segment 53 10
Segment 54 10
Segment 56 10
Segment 57 10
Segment 58 10
Segment 59 11
Segment 60 11
Segment 61 11
Segment 62 11
Segment 63 11

Segment 1

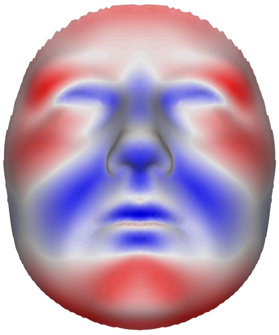

Endophenotypic trait in segment 1. The normal displacement (displacement in the direction locally normal to the facial surface) in each quasi-landmark going from the control to unaffected parent phenotype in segment 1. Blue, inward depression; red, outward protrusion.

Segment 2

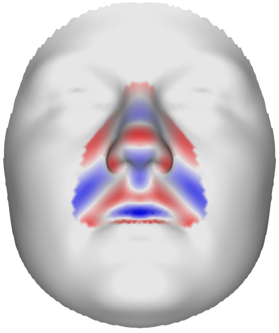

Endophenotypic trait in segment 2. The normal displacement (displacement in the direction locally normal to the facial surface) in each quasi-landmark going from the control to unaffected parent phenotype in segment 2. Blue, inward depression; red, outward protrusion.

Segment 3

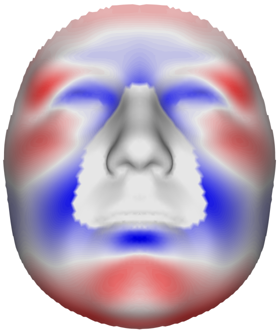

Endophenotypic trait in segment 3. The normal displacement (displacement in the direction locally normal to the facial surface) in each quasi-landmark going from the control to unaffected parent phenotype in segment 3. Blue, inward depression; red, outward protrusion.

Segment 4

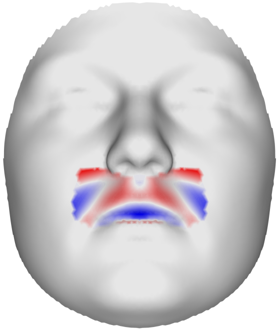

Endophenotypic trait in segment 4. The normal displacement (displacement in the direction locally normal to the facial surface) in each quasi-landmark going from the control to unaffected parent phenotype in segment 4. Blue, inward depression; red, outward protrusion.

Segment 5

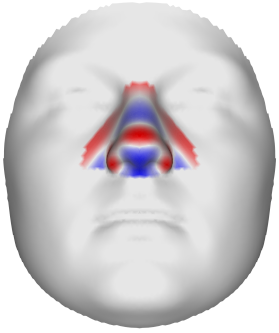

Endophenotypic trait in segment 5. The normal displacement (displacement in the direction locally normal to the facial surface) in each quasi-landmark going from the control to unaffected parent phenotype in segment 5. Blue, inward depression; red, outward protrusion.

Segment 6

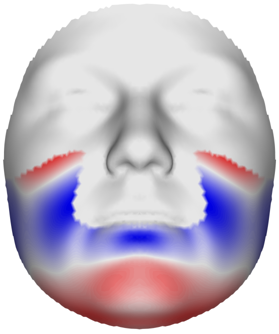

Endophenotypic trait in segment 6. The normal displacement (displacement in the direction locally normal to the facial surface) in each quasi-landmark going from the control to unaffected parent phenotype in segment 6. Blue, inward depression; red, outward protrusion.

Segment 7

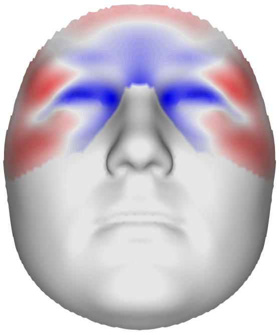

Endophenotypic trait in segment 7. The normal displacement (displacement in the direction locally normal to the facial surface) in each quasi-landmark going from the control to unaffected parent phenotype in segment 7. Blue, inward depression; red, outward protrusion.

Segment 8

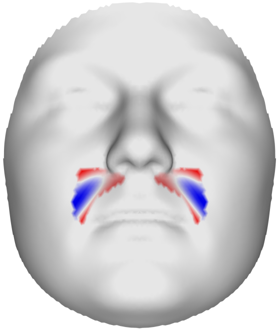

Endophenotypic trait in segment 8. The normal displacement (displacement in the direction locally normal to the facial surface) in each quasi-landmark going from the control to unaffected parent phenotype in segment 8. Blue, inward depression; red, outward protrusion.

Segment 9

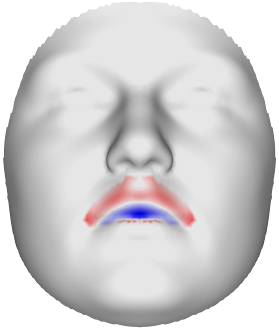

Endophenotypic trait in segment 9. The normal displacement (displacement in the direction locally normal to the facial surface) in each quasi-landmark going from the control to unaffected parent phenotype in segment 9. Blue, inward depression; red, outward protrusion.

Segment 10

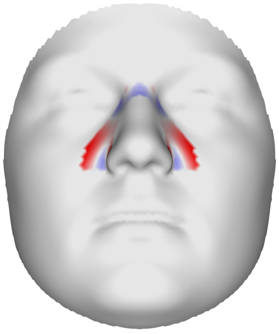

Endophenotypic trait in segment 10. The normal displacement (displacement in the direction locally normal to the facial surface) in each quasi-landmark going from the control to unaffected parent phenotype in segment 10. Blue, inward depression; red, outward protrusion.

Segment 11

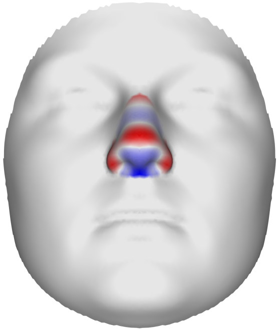

Endophenotypic trait in segment 11. The normal displacement (displacement in the direction locally normal to the facial surface) in each quasi-landmark going from the control to unaffected parent phenotype in segment 11. Blue, inward depression; red, outward protrusion.

Segment 12

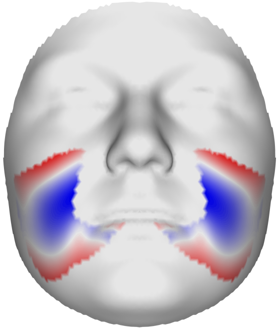

Endophenotypic trait in segment 12. The normal displacement (displacement in the direction locally normal to the facial surface) in each quasi-landmark going from the control to unaffected parent phenotype in segment 12. Blue, inward depression; red, outward protrusion.

Segment 13

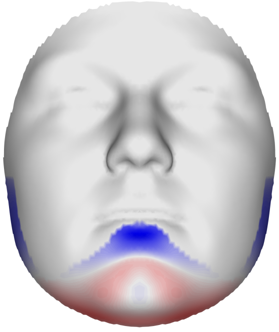

Endophenotypic trait in segment 13. The normal displacement (displacement in the direction locally normal to the facial surface) in each quasi-landmark going from the control to unaffected parent phenotype in segment 13. Blue, inward depression; red, outward protrusion.

Segment 14

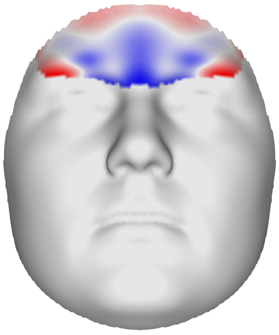

Endophenotypic trait in segment 14. The normal displacement (displacement in the direction locally normal to the facial surface) in each quasi-landmark going from the control to unaffected parent phenotype in segment 14. Blue, inward depression; red, outward protrusion.

Segment 15

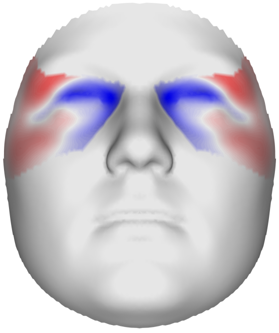

Endophenotypic trait in segment 15. The normal displacement (displacement in the direction locally normal to the facial surface) in each quasi-landmark going from the control to unaffected parent phenotype in segment 15. Blue, inward depression; red, outward protrusion.

Segment 16

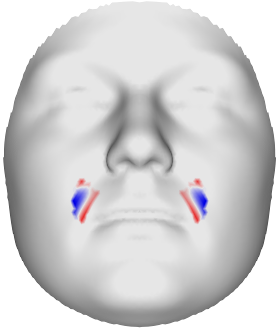

Endophenotypic trait in segment 16. The normal displacement (displacement in the direction locally normal to the facial surface) in each quasi-landmark going from the control to unaffected parent phenotype in segment 16. Blue, inward depression; red, outward protrusion.

Segment 17

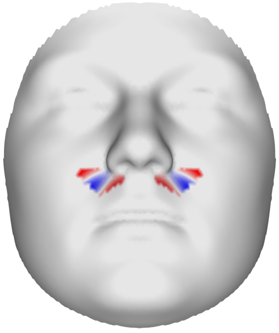

Endophenotypic trait in segment 17. The normal displacement (displacement in the direction locally normal to the facial surface) in each quasi-landmark going from the control to unaffected parent phenotype in segment 17. Blue, inward depression; red, outward protrusion.

Segment 18

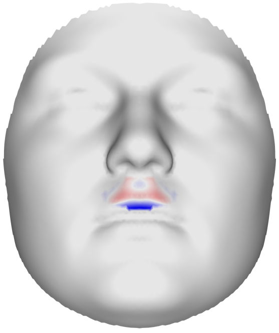

Endophenotypic trait in segment 18. The normal displacement (displacement in the direction locally normal to the facial surface) in each quasi-landmark going from the control to unaffected parent phenotype in segment 18. Blue, inward depression; red, outward protrusion.

Segment 19

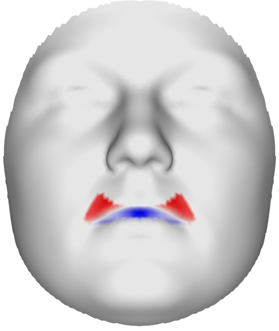

Endophenotypic trait in segment 19. The normal displacement (displacement in the direction locally normal to the facial surface) in each quasi-landmark going from the control to unaffected parent phenotype in segment 19. Blue, inward depression; red, outward protrusion.

Segment 20

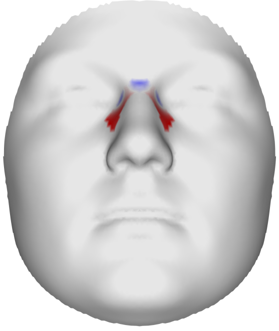

Endophenotypic trait in segment 20. The normal displacement (displacement in the direction locally normal to the facial surface) in each quasi-landmark going from the control to unaffected parent phenotype in segment 20. Blue, inward depression; red, outward protrusion.

Segment 22

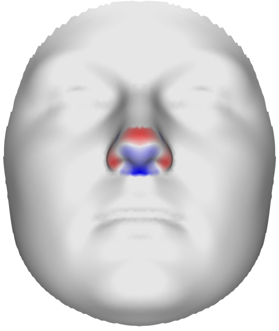

Endophenotypic trait in segment 22. The normal displacement (displacement in the direction locally normal to the facial surface) in each quasi-landmark going from the control to unaffected parent phenotype in segment 22. Blue, inward depression; red, outward protrusion.

Segment 23

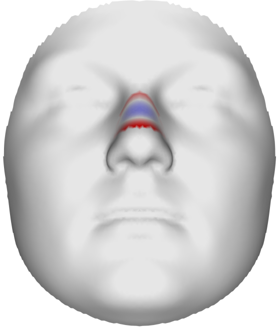

Endophenotypic trait in segment 23. The normal displacement (displacement in the direction locally normal to the facial surface) in each quasi-landmark going from the control to unaffected parent phenotype in segment 23. Blue, inward depression; red, outward protrusion.

Segment 24

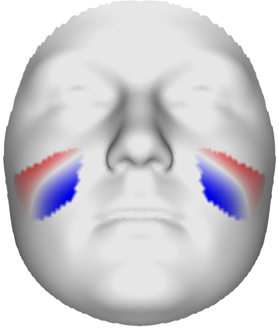

Endophenotypic trait in segment 24. The normal displacement (displacement in the direction locally normal to the facial surface) in each quasi-landmark going from the control to unaffected parent phenotype in segment 24. Blue, inward depression; red, outward protrusion.

Segment 25

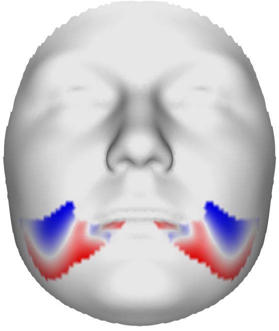

Endophenotypic trait in segment 25. The normal displacement (displacement in the direction locally normal to the facial surface) in each quasi-landmark going from the control to unaffected parent phenotype in segment 25. Blue, inward depression; red, outward protrusion.

Segment 26

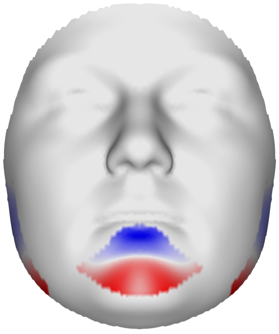

Endophenotypic trait in segment 26. The normal displacement (displacement in the direction locally normal to the facial surface) in each quasi-landmark going from the control to unaffected parent phenotype in segment 26. Blue, inward depression; red, outward protrusion.

Segment 28

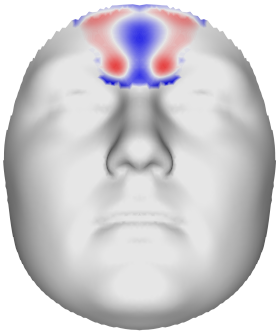

Endophenotypic trait in segment 28. The normal displacement (displacement in the direction locally normal to the facial surface) in each quasi-landmark going from the control to unaffected parent phenotype in segment 28. Blue, inward depression; red, outward protrusion.

Segment 29

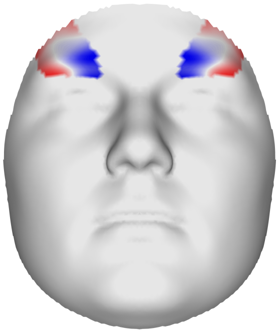

Endophenotypic trait in segment 29. The normal displacement (displacement in the direction locally normal to the facial surface) in each quasi-landmark going from the control to unaffected parent phenotype in segment 29. Blue, inward depression; red, outward protrusion.

Segment 30

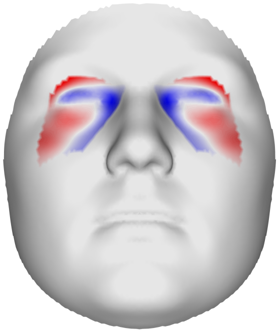

Endophenotypic trait in segment 30. The normal displacement (displacement in the direction locally normal to the facial surface) in each quasi-landmark going from the control to unaffected parent phenotype in segment 30. Blue, inward depression; red, outward protrusion.

Segment 31

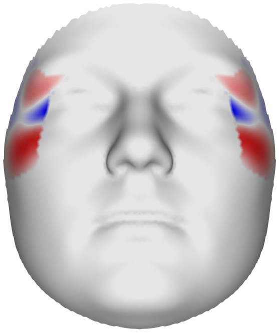

Endophenotypic trait in segment 31. The normal displacement (displacement in the direction locally normal to the facial surface) in each quasi-landmark going from the control to unaffected parent phenotype in segment 31. Blue, inward depression; red, outward protrusion.

Segment 33

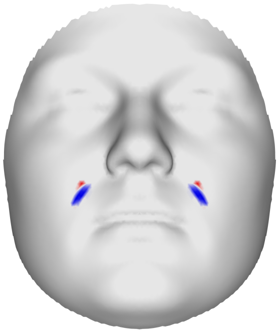

Endophenotypic trait in segment 33. The normal displacement (displacement in the direction locally normal to the facial surface) in each quasi-landmark going from the control to unaffected parent phenotype in segment 33. Blue, inward depression; red, outward protrusion.

Segment 34

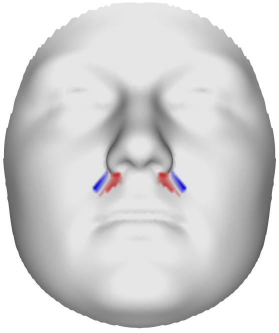

Endophenotypic trait in segment 34. The normal displacement (displacement in the direction locally normal to the facial surface) in each quasi-landmark going from the control to unaffected parent phenotype in segment 34. Blue, inward depression; red, outward protrusion.

Segment 35

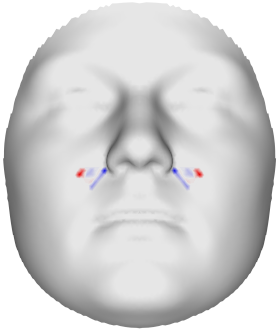

Endophenotypic trait in segment 35. The normal displacement (displacement in the direction locally normal to the facial surface) in each quasi-landmark going from the control to unaffected parent phenotype in segment 35. Blue, inward depression; red, outward protrusion.

Segment 36

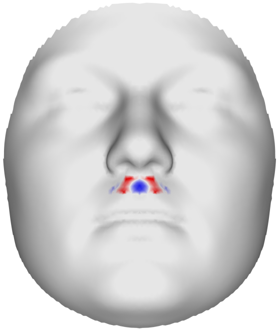

Endophenotypic trait in segment 36. The normal displacement (displacement in the direction locally normal to the facial surface) in each quasi-landmark going from the control to unaffected parent phenotype in segment 36. Blue, inward depression; red, outward protrusion.

Segment 37

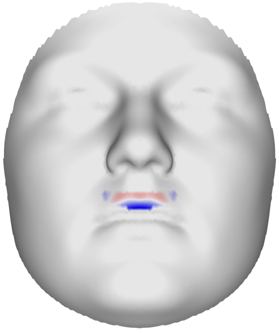

Endophenotypic trait in segment 37. The normal displacement (displacement in the direction locally normal to the facial surface) in each quasi-landmark going from the control to unaffected parent phenotype in segment 37. Blue, inward depression; red, outward protrusion.

Segment 38

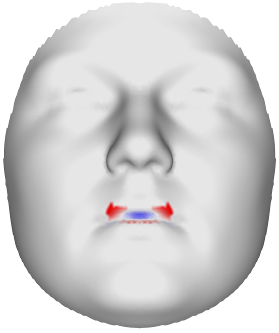

Endophenotypic trait in segment 38. The normal displacement (displacement in the direction locally normal to the facial surface) in each quasi-landmark going from the control to unaffected parent phenotype in segment 38. Blue, inward depression; red, outward protrusion.

Segment 39

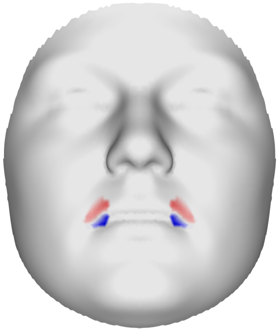

Endophenotypic trait in segment 39. The normal displacement (displacement in the direction locally normal to the facial surface) in each quasi-landmark going from the control to unaffected parent phenotype in segment 39. Blue, inward depression; red, outward protrusion.

Segment 40

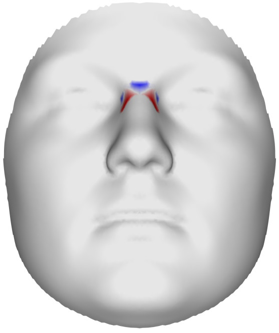

Endophenotypic trait in segment 40. The normal displacement (displacement in the direction locally normal to the facial surface) in each quasi-landmark going from the control to unaffected parent phenotype in segment 40. Blue, inward depression; red, outward protrusion.

Segment 41

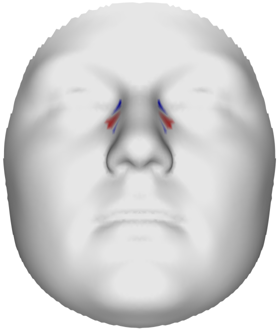

Endophenotypic trait in segment 41. The normal displacement (displacement in the direction locally normal to the facial surface) in each quasi-landmark going from the control to unaffected parent phenotype in segment 41. Blue, inward depression; red, outward protrusion.

Segment 42

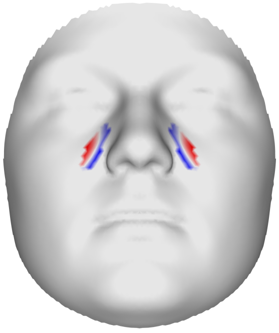

Endophenotypic trait in segment 42. The normal displacement (displacement in the direction locally normal to the facial surface) in each quasi-landmark going from the control to unaffected parent phenotype in segment 42. Blue, inward depression; red, outward protrusion.

Segment 43

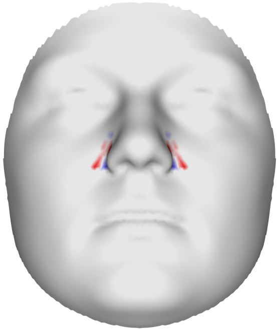

Endophenotypic trait in segment 43. The normal displacement (displacement in the direction locally normal to the facial surface) in each quasi-landmark going from the control to unaffected parent phenotype in segment 43. Blue, inward depression; red, outward protrusion.

Segment 44

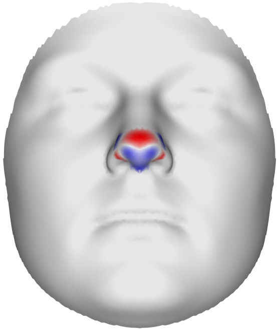

Endophenotypic trait in segment 44. The normal displacement (displacement in the direction locally normal to the facial surface) in each quasi-landmark going from the control to unaffected parent phenotype in segment 44. Blue, inward depression; red, outward protrusion.

Segment 45

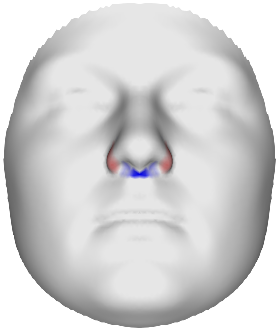

Endophenotypic trait in segment 45. The normal displacement (displacement in the direction locally normal to the facial surface) in each quasi-landmark going from the control to unaffected parent phenotype in segment 45. Blue, inward depression; red, outward protrusion.

Segment 46

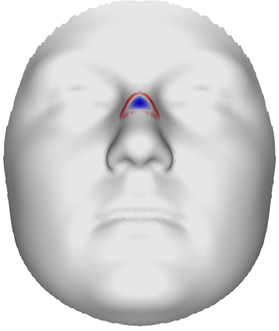

Endophenotypic trait in segment 46. The normal displacement (displacement in the direction locally normal to the facial surface) in each quasi-landmark going from the control to unaffected parent phenotype in segment 46. Blue, inward depression; red, outward protrusion.

Segment 47

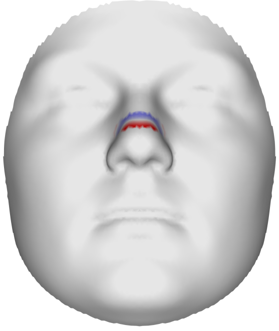

Endophenotypic trait in segment 47. The normal displacement (displacement in the direction locally normal to the facial surface) in each quasi-landmark going from the control to unaffected parent phenotype in segment 47. Blue, inward depression; red, outward protrusion.

Segment 48

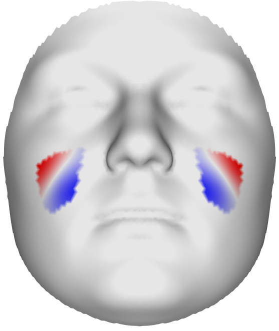

Endophenotypic trait in segment 48. The normal displacement (displacement in the direction locally normal to the facial surface) in each quasi-landmark going from the control to unaffected parent phenotype in segment 48. Blue, inward depression; red, outward protrusion.

Segment 49

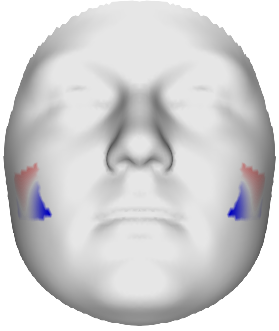

Endophenotypic trait in segment 49. The normal displacement (displacement in the direction locally normal to the facial surface) in each quasi-landmark going from the control to unaffected parent phenotype in segment 49. Blue, inward depression; red, outward protrusion.

Segment 50

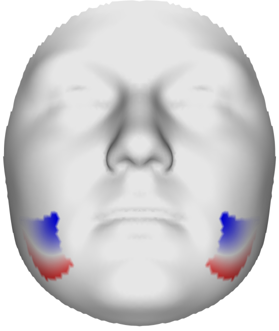

Endophenotypic trait in segment 50. The normal displacement (displacement in the direction locally normal to the facial surface) in each quasi-landmark going from the control to unaffected parent phenotype in segment 50. Blue, inward depression; red, outward protrusion.

Segment 51

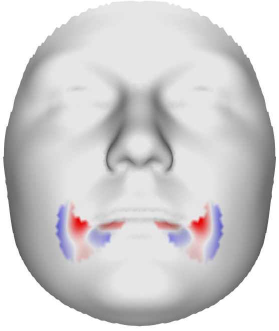

Endophenotypic trait in segment 51. The normal displacement (displacement in the direction locally normal to the facial surface) in each quasi-landmark going from the control to unaffected parent phenotype in segment 51. Blue, inward depression; red, outward protrusion.

Segment 52

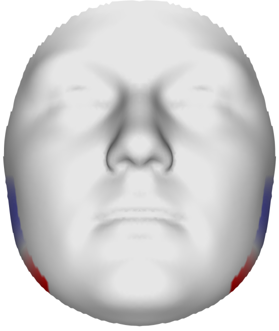

Endophenotypic trait in segment 52. The normal displacement (displacement in the direction locally normal to the facial surface) in each quasi-landmark going from the control to unaffected parent phenotype in segment 52. Blue, inward depression; red, outward protrusion.

Segment 53

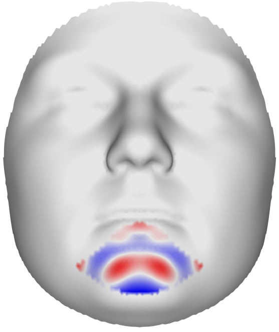

Endophenotypic trait in segment 53. The normal displacement (displacement in the direction locally normal to the facial surface) in each quasi-landmark going from the control to unaffected parent phenotype in segment 53. Blue, inward depression; red, outward protrusion.

Segment 54

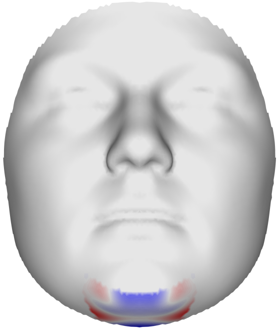

Endophenotypic trait in segment 54. The normal displacement (displacement in the direction locally normal to the facial surface) in each quasi-landmark going from the control to unaffected parent phenotype in segment 54. Blue, inward depression; red, outward protrusion.

Segment 56

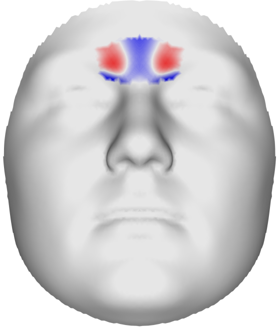

Endophenotypic trait in segment 56. The normal displacement (displacement in the direction locally normal to the facial surface) in each quasi-landmark going from the control to unaffected parent phenotype in segment 56. Blue, inward depression; red, outward protrusion.

Segment 57

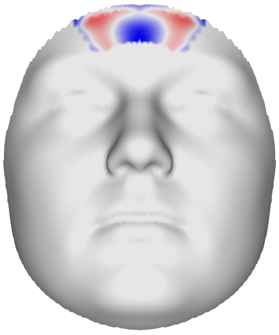

Endophenotypic trait in segment 57. The normal displacement (displacement in the direction locally normal to the facial surface) in each quasi-landmark going from the control to unaffected parent phenotype in segment 57. Blue, inward depression; red, outward protrusion.

Segment 58

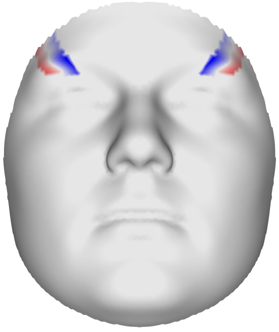

Endophenotypic trait in segment 58. The normal displacement (displacement in the direction locally normal to the facial surface) in each quasi-landmark going from the control to unaffected parent phenotype in segment 58. Blue, inward depression; red, outward protrusion.

Segment 59

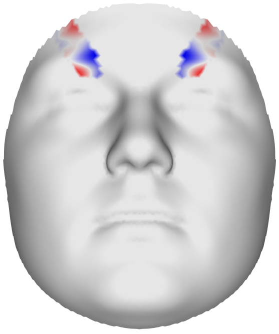

Endophenotypic trait in segment 59. The normal displacement (displacement in the direction locally normal to the facial surface) in each quasi-landmark going from the control to unaffected parent phenotype in segment 59. Blue, inward depression; red, outward protrusion.

Segment 60

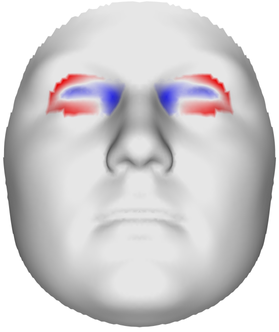

Endophenotypic trait in segment 60. The normal displacement (displacement in the direction locally normal to the facial surface) in each quasi-landmark going from the control to unaffected parent phenotype in segment 60. Blue, inward depression; red, outward protrusion.

Segment 61

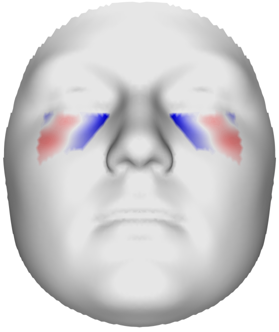

Endophenotypic trait in segment 61. The normal displacement (displacement in the direction locally normal to the facial surface) in each quasi-landmark going from the control to unaffected parent phenotype in segment 61. Blue, inward depression; red, outward protrusion.

Segment 62

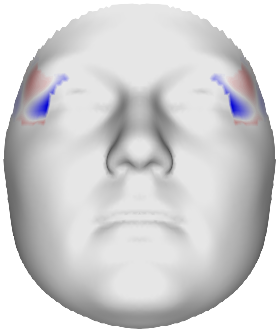

Endophenotypic trait in segment 62. The normal displacement (displacement in the direction locally normal to the facial surface) in each quasi-landmark going from the control to unaffected parent phenotype in segment 62. Blue, inward depression; red, outward protrusion.

Segment 63

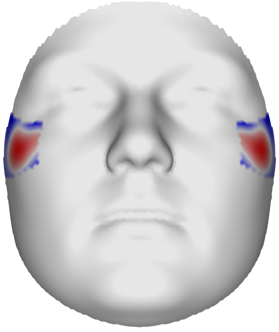

Endophenotypic trait in segment 63. The normal displacement (displacement in the direction locally normal to the facial surface) in each quasi-landmark going from the control to unaffected parent phenotype in segment 63. Blue, inward depression; red, outward protrusion.
